# Co‐developing a common glossary with stakeholders for engagement on new genetic approaches for malaria control in a local African setting

**DOI:** 10.1186/s12936-020-03577-y

**Published:** 2021-01-21

**Authors:** Elinor Chemonges Wanyama, Bakara Dicko, Lea Pare Toe, Mamadou B. Coulibaly, Nourou Barry, Korotimi Bayala Traore, Abdoulaye Diabate, Mouhamed Drabo, Jonathan K. Kayondo, Souleymane Kekele, Souleymane Kodio, Anselme Dinyiri Ky, Richard Ronny Linga, Emmanuel Magala, Wilfrid Ihibna Meda, Solome Mukwaya, Annet Namukwaya, Benjamin Robinson, Hatouma Samoura, Kadiatou Sanogo, Delphine Thizy, Fatoumata Traoré

**Affiliations:** 1 Department of Entomology, Uganda Virus Research Institute, Entebbe, Uganda; 2 Malaria Research and Training Center, University of Sciences, Techniques and Technologies of Bamako, Bamako, Mali; 3 Institut de Recherche en Sciences de la Santé, Bobo-Dioulasso, Burkina Faso; 4 Department of Life Sciences, Imperial College London, London, UK; 5 Emerging Ag, Calgary, AB Canada

## Abstract

Stakeholder engagement is an essential pillar for the development of innovative public health interventions, including genetic approaches for malaria vector control. Scientific terminologies are mainly lacking in local languages, yet when research activities involve international partnership, the question of technical jargon and its translation is crucial for effective and meaningful communication with stakeholders. Target Malaria, a not-for-profit research consortium developing innovative genetic approaches to malaria vector control, carried out a linguistic exercise in Mali, Burkina Faso and Uganda to establish the appropriate translation of its key terminology to local languages of sites where the teams operate. While reviewing the literature, there was no commonly agreed approach to establish such glossary of technical terms in local languages of the field sites where Target Malaria operates. Because of its commitment to the value of co-development, Target Malaria decided to apply this principle for the linguistic work and to take the opportunity of this process to empower communities to take part in the dialogue on innovative vector control. The project worked with linguists from other institutions (whether public research ones or private language centre) who developed a first potential glossary in the local language after better understanding the project scientific approach. This initial glossary was then tested during focus groups with community members, which significantly improved the proposed translations by making them more appropriate to the local context and cultural understanding. The stepwise process revealed the complexity and importance of elaborating a common language with communities as well as the imbrication of language with cultural aspects. This exercise demonstrated the strength of a co-development approach with communities and language experts as a way to develop knowledge together and to tailor communication to the audience even in the language used.

## Background

A broad cross-section of international guidelines and reports have identified community engagement activities as an essential part of any public health project seeking to carry out research involving participants in a manner that is inclusive, responsible, and ethical [1–3]. This is particularly true with regards to projects seeking to adopt a co-development approach [4], in which researchers engage in a collaborative process of jointly designing with stakeholders a research pathway and its resultant intervention to reach a common goal [5]. This entails pro-active dialogue at many different project stages and some measure of shared responsibility for decision-making. It, therefore, goes beyond many common relational paradigms between researchers and stakeholders centred on sharing information and seeking acceptance [1, 6].

These issues are particularly relevant to international research partnerships, in which there is a high likelihood of disparities between participants with regards to perceptions, access to resources, and scientific literacy levels [7]. In these contexts, language barriers can present some of the most significant obstacles to carrying out effective community engagement [8, 9] This is especially true when the research involves new technologies and techniques, for which established consensus terminology may not yet exist in all languages [10]. This proved to be the case for Target Malaria.

### Introduction to Target Malaria

Target Malaria is an international not-for-profit research consortium comprised of research institutions from North America, Western Europe, and sub-Saharan Africa, including teams at four partner institutions in Burkina Faso, Mali, Uganda, and Ghana. Target Malaria is seeking to develop and share a gene-drive based technology to reduce the population of malaria vectors which will complement current and emerging approaches and thereby reduce transmission of the disease in sub-Saharan Africa [11]. With approximately 228 million cases of infections and nearly half a million deaths registered in 2018, malaria remains a priority public health problem, with Africa suffering by far the greatest burden [12]. Given the stalled progress in reducing the incidence of the disease over the period 2016–2018, current methods of combatting malaria will not be enough to allow the world to meet its commitment to controlling the disease under the United Nations Sustainable Development Goal 3 on health (“Ensure healthy lives and promote well-being for all at all ages”) and its specific target “By 2030, end the epidemics of AIDS, tuberculosis, malaria and neglected tropical diseases and combat hepatitis, water-borne diseases and other communicable diseases” [13]. There is, therefore a need for innovative new tools to complement the already existing ones [14, 15].

Target Malaria follows a phased approach in the development of its technology, with gene drive mosquitoes being the ultimate phase as a self-sustaining strain able to spread the modification to the target population. Preceding phases include non-gene drive strains of mosquitoes that are therefore self-limiting. Those mosquitoes are genetically modified, but the modification is not passed at a preferential rate to the progeny and does not persist in the environment.

The project has committed to a co-development approach [16] with local communities and stakeholders (in addition to the co-development between researchers of different backgrounds), as a means of ensuring their concerns and expectations are taken into account for project activities, and that the future technology responds to their actual needs [4]. To achieve this, engagement and outreach efforts must take place in local languages, without which engagement could not be considered meaningful [17].

To do this, it was necessary to develop glossaries translating critical terms related to genetic modification, gene drives, gene editing, entomology, field evaluation, and other relevant aspects of the project into local languages of the field sites where the project has activities, in a manner appropriate to, and accessible for, all local stakeholders. This enabled the project to create consistency in communication relating to scientific terminology, to improve stakeholder understanding of the project activities and to ensure that any eventual consent (at individual level) and acceptance (at community level) are effectively informed.

### Literature review

The need for local language tools and the challenges inherent in ensuring that they can communicate new or complex scientific concepts to stakeholders with widely varying degrees of literacy and knowledge is extensively documented in the field of stakeholder engagement for medical research [8, 18–24]. In the sub-Saharan African Region, documented efforts to develop these tools have mostly been published in relation to research dealing with malaria, HIV, and especially genetic and genomics studies [25].

Among the most detailed and instructive of these is the experience of the KEMRI-Wellcome Trust Research Programme in Kilifi, Kenya [7], which described a process by which researchers translated informed consent forms, originally drafted in English, into Kiswahili, through workshopping and conceptual elaboration, and highlighted the challenges that arose during these activities, and those which yet remain to be overcome. Their analysis, however, remained highly context-specific, and they did not attempt to distil their findings into more broadly applicable good practices, even if such exercises would always need to be tailored for a particular context. Also of particular value are the writings of Traore et al. [26] and Tindana et al. [10] on the process of collecting and analysing the views of MalariaGEN participants in Mali and Ghana. This entailed the development of interview guides in French and in Bamanankan. Traore et al. presents the lengthy process through which the Bamanankan versions were produced, involving expert translators and support from the National Institute of Local Languages, as well as the iterative exercises after they had been developed, in which interview transcripts were back-translated and compared to ensure consistency and clarity in the terminology the research team used. This paper highlights the fact that despite these resources, engagement was not straightforward, and confusion and misconceptions remained due to conceptual barriers of understanding among stakeholders.

There are many other documented cases of the development of local language materials to facilitate stakeholder engagement in settings in which substantial conceptual and linguistic barriers exist. These include HIV prevention trials in India, Thailand, South Africa and Canada [27], informed consent processes in Ghana [28, 29] and vaccine trials in Africa [30] with regards to several African case studies. However, publications to date do not provide a detailed discussion of the ways in which the development of these tools may be carried out, nor attempt to draw up a normative framework of good practices on the basis of these experiences.

### Scope of the case study

Hence, as the project was reflecting on its process and what it has achieved, it appeared that there would be a value in analysing the specificities of the co-development approach taken by the teams to establish these glossaries in the various local languages. This not only provides an example of developing valuable resources for any future related research in comparable socio-linguistic contexts but also about how the process of doing so can be part of the engagement itself. This case study is therefore, an attempt to address the relative paucity of well-documented examples of linguistic work informing engagement in international public health collaborative research [1, 27].

## Methodology

This process originated from a call from the Target Malaria global stakeholder engagement team proposing specific funding for teams interested in taking a systematic approach to developing a glossary for their engagement work. This was part of the general support from the global team to the partner country teams, trying to identify challenges faced in the implementation of engagement and to address those. The overall objective of this process was to support meaningful engagement by having a consistent usage of the local language used by stakeholders. Each partner organization in Burkina Faso, Mali and Uganda developed its own proposal to reach this without a common methodological framework at the inception. However, there are clear commonalities in those approaches, which are presented in this "Methodology" section, along with their differences.

### Partnership with linguistic experts

In the three countries, Target Malaria teams decided to establish a partnership with linguistic experts. The partners were selected based on their knowledge about local languages as well as their experience in working on new terminology. There were no specific criteria about being a public or private institution, and the selection was based on their skills and experience. In the case of Mali and Burkina Faso, they choose to work with public institutes’ experts. In Mali, the partner selected was the National Directorate for Non-Formal Education and National Languages (DNENF-LN), a government institution that plays a critical role in the codification of local languages and their transmission. In Burkina, the experts were linguists from the Institute of Social Sciences (INSS), which is the main institute for national languages in country. In Uganda, the expert was from a private company (the Kampala Language Centre) specialized in language teaching and translation services. This group was chosen because the project had worked with them for the interpretation of meetings that took place in Uganda and felt they had a good knowledge of the project and its technical language.

### Stepwise approach

**Fig. 1:**
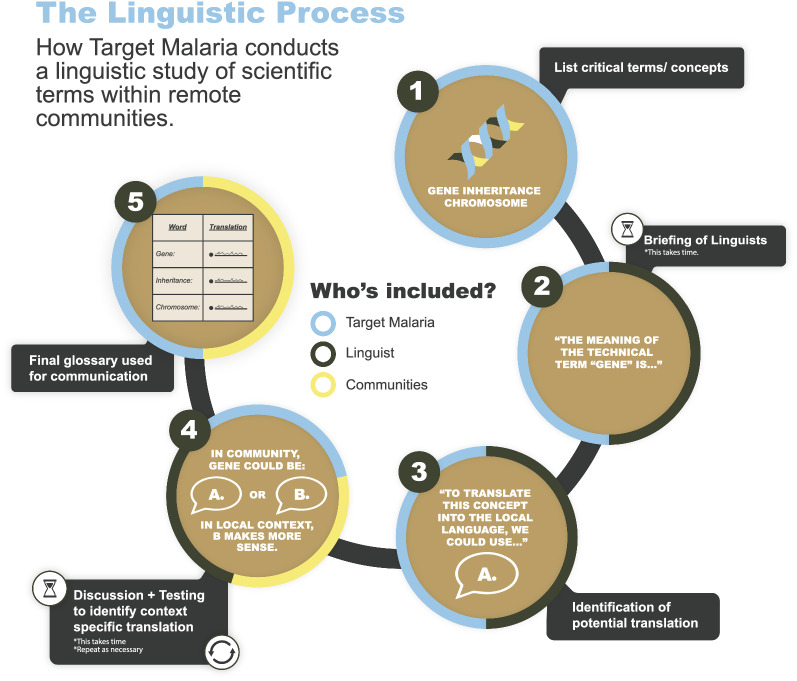
A step-by-step linguistic process

The process followed a stepwise approach, with five main steps (Fig. 1). Once the partner was selected, the first step for the teams was to establish a list of the critical terms or concept that required inclusion in the glossary exercise. These terms cover a number of thematic topics from genetics (gene, chromosome, DNA), to entomology (mosquito, larvae, collection, swarming), laboratory (containment, insectary, biosecurity), through more common engagement language (consent, engagement, community acceptance).

As a second step, the linguists were briefed on the project to ensure that they understood what the technology was, the science behind it, and the approach. This was done in meetings with both the stakeholder engagement teams and the scientific teams (field entomologists and insectary staff). This was a time-consuming step as linguists were not necessarily very used to these concepts or terminology and their understanding was critical to ensure that the terms chosen in the target language would be accurate.

As a third step, the linguists held meetings with the project researchers to select the most appropriate translation for each word, by checking the scientific accuracy of the terminology proposed. From this, an initial glossary was created for further elaboration and discussion with stakeholders. In the case of Burkina Faso, initial translations had been suggested between the second and third step, but the meeting was necessary as the original translations showed some misunderstanding of the terminology used.

The fourth step involved focus groups with members of the community. These members were selected by the village leadership based on their good knowledge of the language, their eloquence and the fact they were considered trustworthy by the community. The number varied between 5 and 8 people depending on the village, and men and women were represented in these focus groups. The focus groups involved a deliberative consultation between the project researchers (stakeholder engagement team members), the linguists, and the community representatives. During those discussions, the translations were tested to see how they were understood, and if the community wanted to adjust them to improve the clarity. In the case of Burkina Faso, in the following weeks and months of engagement, the terminology was further refined with the inputs from the community in a more informal way with stakeholders proposing improvements. In countries where the testing took place in several villages (Uganda and Mali), the inputs were integrated at each stage, so the learnings from the first village were further tested in the second village and so on. This step was considered the most time-consuming because of the coordination needed to find a time that would suit for all the different stakeholders to carry out the testing exercise.

From this, as a fifth step, a glossary was elaborated in the three countries and shared with other team members of the project. In the case of Mali, this was followed by a proposal to publish this glossary so that other researchers or users could benefit from this work. Similar work is being considered in Burkina Faso.

### Site selection

The countries where Target Malaria operates in Africa are multilingual. The linguistic maps vary substantially between countries. In Uganda, Luganda is a wide-spread language in the region around Entebbe and on the Lake Victoria islands, where the project operates. There are no other languages in the area; however, there are several dialects deriving from this common language and with local specificities. Nevertheless, Luganda is a commonly understood language in the area. The team choose three villages to test the glossary: Buliro, Kituntu located in Mpigi district, and Kitamba located in Masaka district. Those villages where the glossary was pretested were not part of the project sites where activities take place but were in the Luganda speaking catchment area. This is due to the coincidence between this activity and a scouting process for new potential field sites on the mainland part of Uganda, and as such, the project did not have a long-standing relationship with communities. In those areas where the project operated at the time of the study, Luganda was the dialect common to the whole population of the area, without differences related to ethnic minorities. In Mali, Bamanankan is the dominant language in the area where the project operates; there are no other languages spoken in the area, though they exist in other parts of the country. At the time, the project was working in four villages (Ouassorola, Kababougou, Sogolombougou and Tieneguebougou) of the Koulikoro region, and did the testing in those villages. In those there are no migrant populations that would speak another language. The situation in Burkina is different, with far more diversity of languages within the area, partially due to migrations between regions. Within one village, several languages can be used. However, in the villages where the project operates, the community uniformly uses Dioula language to communicate between people of different native languages, which is consistent with the fact that Dioula is the common language of the Bobo-Dioulasso province. The Dioula language does not vary between locations, and the residents are culturally quite similar. Thus, the team and expert linguists decided to carry out the testing in one location, the village of Bana.

## Results

### Timing and context of the linguistic work

The three countries implemented this research in different contexts, reflecting the degree of research progress in each country. In Uganda, this took place while the team was scouting for new field sites, and thus the research was done in locations with no prior knowledge of the project. In Mali, the team had been doing routine field entomology collection in the locations, but had not yet started engaging about the first strain of genetically modified mosquitoes. In Burkina, the engagement about the first strain of sterile male mosquitoes had been initiated using the team’s translation of the key terminology. These differences allowed comparison to some extent of the impact of timing of the glossary elaboration. In the case of Burkina, early engagement presented linguistic challenges. When explaining the sterility of the mosquito causing the absence of viable eggs fertilized by these mosquitoes, the expression used was /sosso kô bôni/, (literally: sosso = mosquito; kô = literally back; bôni = remove; kô is often referring to virility, and in particular when associated with bôni it refers to sexual impotence) alluding to castration as it echoed the community’s experience of domestic husbandry. This impacted understanding as feedback collected from engagement meetings showed that community members envisaged this intervention as the physical castration of male mosquitoes and did not originally understand that this was the effect of a genetic modification nor that those mosquitoes would have mating events with females. Based on the concept of gene ‘Fɛɛn fitini dɔ lo, a be farisogo kɔnɔ, a be yɛlɛmani don farisogo ra’ (literally something that is small, inside the body and that creates changes in the body), the concept of genetic modification was developed as “Farisogo cogo yɛlɛmanin’ (the change of the way the body is). The result of the co-development process was to use: « Soso cèman min cogo (~ dancogo) yèlèmana, walisa n’a jènna ni soso musoman ye, musoman be faan la, nga, faan nunu te tôtô » meaning « male mosquitoes that have received a genetic modification so that once they mate with females, those will lay eggs but they will not be viable ». In Burkina Faso, the linguistic work was envisaged as a tool to help address these misconceptions and improve the clarity of the message delivered. However, the original term remained in the community, and the team had to make additional efforts to correct this through continuous engagement re-explaining the process. While in Mali, where the engagement on this topic had not been initiated, the linguistic work helped give confidence to the stakeholder engagement team about their ability to communicate those complex terms in a meaningful and culturally appropriate way from the beginning. In Uganda, this process helped the team introduce its work in a more understandable way to new communities. This could have contributed, all things being equal, to field entomology improvements as the communities were able to help the field entomology team identify mosquito swarm sites, with an improved translation of the word “swarm”.

### The complexity of translation

In Burkina Faso, the team had asked the linguists to translate the terms from the list they had drawn up in French. Following this, there was a number of interactions between the linguists and the team members, culminating in a meeting with the linguists where the scientists and engagement team met to amend some of the proposed translations based on their experience and knowledge. In Mali and Uganda, the teams developed the translations with the linguists after ensuring that everyone had the same understanding of the terminology in the original language (French in Mali and English in Uganda). In Mali, this process took the form of a 2-day workshop organized jointly by Target Malaria and the DNENF-LN, bringing together both researchers and linguistic experts. In all countries, those explanations did not only focus on the word and concept but included a broader explanation of the project science. For instance, the expression “sterile male mosquito” was not only explained as a male mosquito unable to have progeny but a simplified explanation of the genetic process and the fact that this mosquito was able to produce sperm but that the eggs fertilized with this sperm would be non-viable and thus that no progeny would emerge from such mating [31], was explained. That revealed the importance of ensuring alignment of the different partners on the meaning of the terminology in the original language and of a basic understanding of the concepts used.

Once the linguists and researchers had a common language in French or English, the translation was developed through a dialogue between the two expert groups. This process rarely involved direct translation of the terms, as they did not necessarily exist as such in the local language, but rather a description of the underlying concept. For instance, the word gene was not translated directly in Luganda but rather through the expression “Endaga butonde esokerwako mu kutondawo ekintu ekirina obulamu” (meaning literally a unit of a DNA that is responsible to bring forth life). Interestingly the language already had an expression for DNA “Endaga butonde”, which is more commonly used and understood by the communities. Similarly, in Dioula, the concept of biosafety was translated by “Danfɛnw latanganan fɛɛrɛw - Danfɛnw faratikow ɲatigɛli” (meaning literally protection measures for living beings—a risk or danger prevention related to living beings).

In those meetings, the linguists and the team members aimed at finding a consensus on each translation either by finding a direct word translation or by finding an expression that could illustrate this word in the local language. The question of using neologism was discussed between the project team, the experts and the community. In most cases, that option was rules out because there were other ways to translate the terms that were more meaningful for the community—mainly by using different words that described the concept. The rare exception is the term “Koromozomu” in Dioula, because both the experts and the community thought that the alternative would have been too cumbersome and complex. In that case, the neologism is borrowing from the French word “chromosome” and is adapted to the phonetics of Dioula. However, they added an explanation in the local language accompanying this new word “Farisogo yɔrɔnin dɔ lo, a ka dɔgɔ, a ti ye ɲa na, a be ninmanfɛnw cogoya yira, o cɛya walima o musoya” (meaning literally it’s a part or a specific place of the body that is small and that we cannot see with naked eye and that shows the nature of living beings, whether they are male or female).

### Language consistency within the communities

In the three countries, the participants of the focus groups were selected by the community leaders (chairman in Uganda or traditional chief in Mali and Burkina Faso). The main criteria were their good knowledge of the language and their trustworthiness. The concept of trustworthiness in the experience of Target Malaria is usually used by village leadership to ensure that the volunteer proposed will carry out the work in good faith and provide reliable inputs. Both men and women were represented in those focus groups.

While there were discussions between the participants and the linguists and project about different translations of terms, the inputs from the participants were quite consistent, and there was no evidence of strong differences in the use of terms or images to illustrate a concept between men and women, education level. For instance, when comparing the initial translation from the linguists and the final list co-developed with the community, the education level (university degrees) of the researchers did not create a gap in language with the community members. The main differences come from the images used to better explain a concept, which are deeply rooted in the community’s culture or experience. For instance, in the Burkina Faso testing site, the question of gene inheritance was illustrated with albinism trait inheritance as it is well-known in the community. The choice of those references was a greater determinant of differences in the language used between the linguists or the team members and that used by the community than education levels. Interestingly, even when checking the understanding of the new terms developed through this work with other parts of the community who might not share the same native language (which was the case in the village of Bana in Burkina where ethnic and thus linguistic minorities are present), the understanding was consistent.

### The importance of communities’ inputs

In Uganda, six focus group discussions were organized. Both representatives of the project team and the professional linguist participated. The groups were asked to explain what they understood from the translated words, and this helped in deriving appropriate translations for the scientific words. For translated words that were difficult for the groups to understand, the professional linguist and the stakeholder engagement team would explain what they intended to refer to, then the focus group discussion members would give a translation understood by the whole group that would correspond to the explanation. In all countries, participants were questioned about their understanding of the term proposed by the linguist and the team, and were encouraged to propose alternatives when the translation was not clear enough (i.e., when there was a discrepancy between the term’s original meaning and its understanding from stakeholders) or when a clearer translation could be provided integrating elements of the local culture. For instance, the linguists had proposed to use the term “omulangasira” to refer to male sterility. While correct, this term is not current, and both the team and stakeholders did not feel comfortable using it as its meaning is not understood widely. Instead, they chose the term “omugumba”, which is commonly used to refer to sterility in general but that literally means a woman who cannot bear children. This reflects a strong cultural bias on the fact that infertility in the couple is due to women’s sterility, which explains why all kind of sterility are described by a word meant to designate female sterility. In Dioula, the final terms in the glossary often take the form of the description of functions of a given concept, rooted in images that could be drawn from the daily lives of stakeholders. For instance, in the community, the reference to albinism is often used to explain gene inheritance, as this trait is considered as common in the area and community members understand its inheritance. This confirms findings from other studies showing that comparisons are helpful to communicate with community members and to demystify complex concepts [23].

These inputs have clearly improved the glossary and made significant changes, as shown in illustrating the glossary before and after testing for the term “chromosome” or “generation” (Tables 1, 2).

**Table 1:** Translation before and after the focus group discussions with community members in Burkina Faso

Phase of translation	Words/expressionin French	Translation in Dioula	Explanation/example
Before the focus group discussions	Chromosome	Farisogo yɔrɔnin dɔ lo, min be ninmanfɛnw cogoya yira	It's a part of the body that determines the characteristics of living beings
After the focus group discussions	Chromosome	Koromozomu	Farisogo yɔrɔnin dɔ lo, a kadɔgɔ, a ti ye na na, a beninmanfɛnw cogoya yira, ocɛya walima o musoya.(literally: it’s a part or aspecific place of the bodythat is small and that we cannot see with naked eye and that shows the nature of living beings, whether they are male or female)

**Table 2:** Translation before and after the focus group discussions with community members in Mali

Phase of translation	Words/expression in French	Translation in Bamanakan	Literal translation﻿
Before the focus group discussions	Generation	Sɛrɛ	People born at the same moment
After the focus group discussions	Generation	Waatikelen	People born around the same period

### Using the glossary

After the testing, the teams finalized the glossary of terms. The final step was to train other team members on these terms to ensure consistency throughout the project team. This was done with field entomology, insectary and other members of the teams as well as local volunteers who help with mosquito collection.

Overall, the teams have found that having this glossary has helped with engagement as they did not have to come up themselves with potential ways to explain complex scientific concepts but were able to rely on tested terminology. In Burkina Faso, the shift in the terminology to describe a sterile male mosquito (from the original “castrated male” to the new translation meaning “a male mosquito which cannot have progeny”) impacted the understanding of the community and ensured a more informed consent. This consent was more informed because it was clear to community members interviewed that the male was able to produce sperm and mate but that the eggs would not hatch (as observed during the subsequent surveys of community understanding and project audit).

In addition to this, the glossaries were used in subsequent communication tools. For instance, animated videos were developed to explain the field entomology collection methods, and all have voices in local languages. The main benefit from this is that there is a consistency in the message provided, regardless of the team member presenting the information, as the vocabulary is standardized. As those words were validated with the community members, it also increases the understanding of those concepts.

Finally, such project and linguistic work are contributing to the evolution of a language by adding new words (such as Koromozomu in Dioula). The history of HIV and associated stigmas demonstrated that a careful approach is important in those phases of language development as it can impact how research or specific aspects of science are perceived by the communities [32, 33].

## Discussion

### Translation as an iterative process

Before beginning the process of translation from English to Luganda, or French to Dioula or Bamanankan, the Target Malaria teams first sought to universalize as far as possible the concepts under discussion and to make them understandable to the linguist. By doing so, they were already engaging in translation from a highly specific scientific language to a language accessible to non-experts. Although not necessarily desirable in a scientific context, a higher degree of abstraction can be useful in making concepts intelligible to as broad an audience as possible, to create a language that can be common across society, which in return facilitates broader engagement. Any resulting lack of detail or precision must not obscure potential risks or benefits to stakeholders.

The fact that specific terms may not exist in local languages for new technologies or scientific concepts does not preclude the creation of explanatory formulations comprehensible to laypeople (for example metaphorical images or allegorical allusions) [34]. The day-to-day experiences of stakeholders can be drawn upon to inform illustrative images. The translation must take into account not only the transition from one language to another but also a potential transition from one socio-economic or cultural context to another. As such, it is important to check whether translation and explanation developed and tested with a specific community are understood similarly when entering a new community if it does not belong to the same cultural group.

This process highlights the complexity of translation of scientific terminology into languages and culture that might not have a direct translation for those terms but are able to understand those concepts using analogies and images. For a project like Target Malaria working on genetic approaches, there is a recognition that the process of naming (genetic components, attributes of the technology) is complex. What has been observed between French/English and local languages can similarly be observed when translating from scientific English to French. In this perspective, the concept of “gene drive” has proven equally tricky in French as it is in Luganda or Dioula. As there is no direct translation in French, various expressions have been used, whether “forçage génétique” for instance, used in the Convention on Biological Diversity translations [35] or “impulsion génétique” used by the International Union for Conversation of Nature in more recent translations [36]. Those challenges are not unique to Low- and Middle-Income Countries contexts and to some extent, they are inherent to scientific discoveries and the process of developing new knowledge. The process that Target Malaria is going through in local languages is quite similar to those of the early days of genetics and the use of the word “gene” [37]. It can be disconcerting for teams as this requires constant awareness and openness for new word propositions from stakeholders, but it is part of a new terminology establishment process.

### Importance of a multi‐stakeholder co‐development approach

Co-development is one of Target Malaria’s core values [16] and is deeply rooted in an ethical principle to empower directly affected communities to make a decision about this new technology and its evaluation pathway. There are also instrumental reasons for co-development, and the glossary development process is a good example of this.

The project team members had the intellectual knowledge, know-how and field experience to translate the project terminology into local languages—and had been doing so before this process, in particular in Burkina Faso. However, the partnership with national linguists and the communities has proven more efficacious to develop a glossary adapted to the socio-cultural context by integrating communities’ experience into the process and helping the normative process with the linguists’ expertise. This is an excellent example of knowledge engagement [4], where the project co-develops new knowledge—in this case, a new language glossary—with stakeholders. While the communities and the project members are at the heart of this co-development, the addition of the linguistic experts brought a critical dimension to the process. Above all, it contributes to the scaling of the process, by formalizing the glossary produced, giving it a scientific rigour and credibility to allow the project and other researchers to trust that those translations are both adapted to the communities but also following the language rules of syntax for instance. As such, the choice of the linguistic partner needs to take into account the reputation of the institution, their experience and skills to develop such glossary. Reflecting back on the experience, there is an added value in working with a public institution (as it was the case in Mali and Burkina Faso) as this can help amplifying the results by integrating them to the national research on linguistic.

To ensure this co-development approach is successful, it is critical that the project pays attention to the representativeness of different groups, whether gender-based, age-based, or socio-culturally-based. Though in this particular experience, there were no significant differences in the terminology proposed or its understanding based on those criteria, it is crucial to verify this assumption by having differences represented in the testing group, and when appropriate by taking measures for potential differences to be expressed. For instance, in Uganda, that meant having men and women in different groups to allow women to express themselves freely.

In addition, the process needs to establish means of reconciling differing opinions with regards to the meaning, connotations, or implications of terminology that do not unduly privilege the viewpoints of certain experts over others. By taking decisions through consensus, the workshop and the focus groups ensured that participants with many different types of knowledge and experience were able to take part in the development of the glossary on an equal footing.

### Do it early but with some prior knowledge of stakeholders

The example of Burkina Faso and the impact of a changing terminology on understanding calls for early investment in linguistic activities. This requires identifying relevant stakeholders, assessing their potential language gaps related to key terms and concepts, and planning how to address them early in the project implementation process. To manage the risk of misconceptions emerging from inappropriate or incomplete translations, projects need to evaluate what new communication tools, in what languages, will need to be developed, and how, before any significant engagement is carried out. However, there is value in having some prior knowledge about the community dynamics and linguistic challenges, if only to identify what language should be used or the pre-existing knowledge of similar terminology. This will entail some basic engagement activities, and analysis to determine who engagement activities will be targeted at, and what shared conceptualizations and linguistic gaps may exist between them and researchers relevant to the work of the project. This process should ideally be carried out before any research in the field is undertaken and will then need to be revisited as the project progresses and introduces new concepts but also according to the feedback from the communities and what those will reflect about their understanding of the concepts. These activities should be reflected in timelines during the project design phase, and the appropriate allocation of resources for them should be reflected in project budgets. The resources invested for this activity have varied between the different teams, due to different context. In West Africa, the process costed around 2000 USD, while in Uganda cost was around 1000USD—including the fees of the linguist experts and other small costs for the meetings (the cost of the project human resources is not integrated to that as the activity was part of their scope of work). In terms of time allocation, the overall process took in average 2 months of intense work, usually over a period of 5 to 7 months. The most time-consuming parts were the coordination of different stakeholders’ availability (researchers, linguists and communities) as well as the coordination with scientific experts to ensure that the linguists were clearly understanding the concepts to translate. While the whole stakeholder engagement team was mobilized for the study, on a punctual basis, other project members were brought in, such as the field entomology, insectary experts or the Principal Investigators—for instance for meetings with the linguists. When reflecting on the process, the teams establish that the learnings done during this first pilot could easily create some effectiveness in the process and shorten the time spent on it. As an example, the Target Malaria team in Ghana who is implementing some ecological research activities has done a similar glossary work and completed the process within a month. Overall, this process is easily implementable and worth the investment for the multi-years projects but might be very demanding for a single-year project.

## Conclusions

**Fig. 2:**
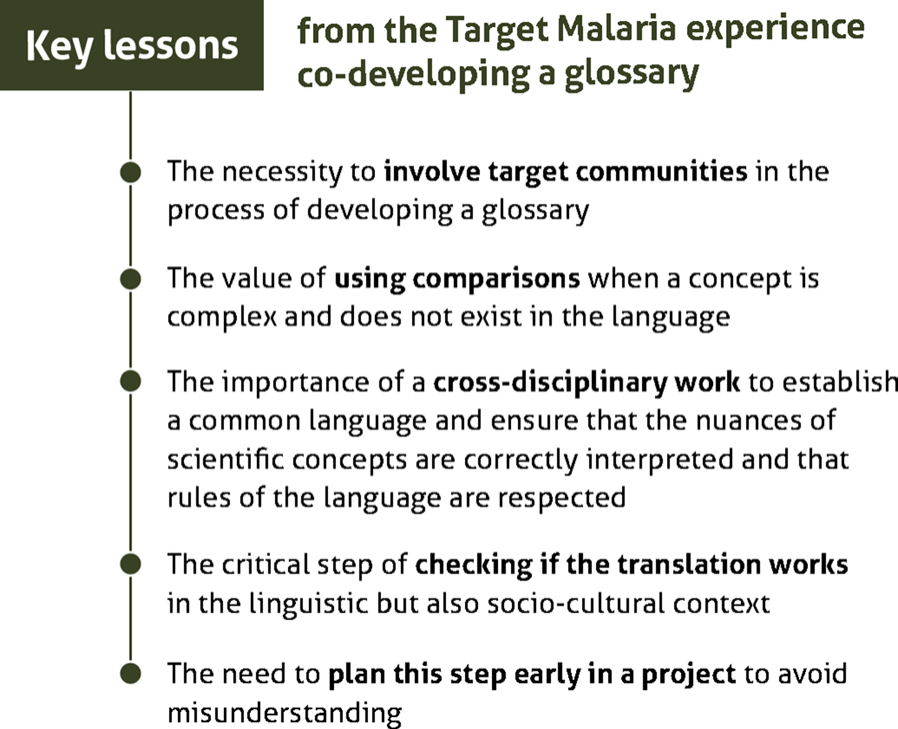
Key lessons

Despite the differences in methodology, these cases have shown some commonalities and learnings that can serve as a useful resource to researchers planning and implementing stakeholder engagement in local languages, and which may contribute to the eventual standardization of good practices in this area (Fig. 2). The Target Malaria teams have found that these normative principles can constitute useful examples in the development of stakeholder engagement tools in local languages, in contexts where significant conceptual barriers or variations in literacy and knowledge levels may be present. Although these examples pertain to research into malaria control in Sub-Saharan Africa, many of these guidelines are broad enough to be useful starting points for researchers in a wide variety of disciplines and regional contexts. Despite this, they are not comprehensive, and the many processes through which local language tools can be effectively developed, and the trade-offs, mutual reinforcements or redundancies they can entail, as well as the contextual appropriateness of each method remain under-studied. This paper represents an early step in synthesizing lessons-learned (Table 2) across a number of case studies to lay the foundations for a general framework of effective engagement in local languages. In addition, with the publication of these glossaries, the project hopes to contribute to a constructive dialogue on genetic approaches in those societies.

However, all the teams noted the limits of this process, which focused on one language at this point. Whether it is to integrate part of the population in new field sites where migrant workers do not share the main language (such as in Uganda) or to better reflect the diversity of languages of the area in particular when considering expanding the engagement to new villages (such as in Burkina Faso), the reproduction of this work in other languages is critical. In West Africa, where the partner institutions have knowledge of other languages, the process could be simplified, and the translation work could start from the Dioula and Bamanankan languages focusing more on the process to adapt it to the culture of those new communities.

Overall, this experience has demonstrated to the project the usefulness of devoting resources to the development of local language terminology and engagement tools early on in the research process. Considering the relative paucity of such efforts, there would be a real value in having a more concerted approach within countries to mobilize resources and efforts towards this aim.
